# Chronic MAP4343 reverses escalated alcohol drinking in a mouse model of alcohol use disorder

**DOI:** 10.1038/s41386-023-01529-z

**Published:** 2023-01-20

**Authors:** Giovana C. Macedo, Max Kreifeldt, Scott P. Goulding, Agbonlahor Okhuarobo, Harpreet Sidhu, Candice Contet

**Affiliations:** 1 Department of Molecular Medicine, The Scripps Research Institute, La Jolla, CA USA; 2 Faculty of Pharmacy, Department of Pharmacology & Toxicology, University of Benin, Benin City, Nigeria

**Keywords:** Addiction, Prefrontal cortex

## Abstract

Alcohol use disorders can be driven by negative reinforcement. Alterations of the microtubule cytoskeleton have been associated with mood regulation in the context of depression. Notably, MAP4343, a pregnenolone derivative known to promote tubulin assembly, has antidepressant properties. In the present study, we tested the hypothesis that MAP4343 may reduce excessive alcohol drinking in a mouse model of alcohol dependence by normalizing affect during withdrawal. Adult male C57BL/6J mice were given limited access to voluntary alcohol drinking and ethanol intake escalation was induced by chronic intermittent ethanol (CIE) vapor inhalation. Chronic, but not acute, administration of MAP4343 reduced ethanol intake and this effect was more pronounced in CIE-exposed mice. There was a complex interaction between the effects of MAP4343 and alcohol on affective behaviors. In the elevated plus maze, chronic MAP4343 tended to increase open-arm exploration in alcohol-naive mice but reduced it in alcohol-withdrawn mice. In the tail suspension test, chronic MAP4343 reduced immobility selectively in Air-exposed alcohol-drinking mice. Finally, chronic MAP4343 countered the plasma corticosterone reduction induced by CIE. Parallel analysis of tubulin post-translational modifications revealed lower α-tubulin acetylation in the medial prefrontal cortex of CIE-withdrawn mice. Altogether, these data support the relevance of microtubules as a therapeutic target for the treatment of AUD.

## Introduction

Alcohol use disorders (AUD) encompass pathological patterns of excessive alcohol drinking, in which the motivation to increase alcohol consumption or relapse after a period of abstinence is often fueled by negative affect [1, 2]. Pharmacological studies seeking to understand and alleviate AUD have traditionally focused on neurotransmitter, neuromodulator, and neuroimmune signaling, with little attention given to structural proteins [3–5].

In recent years, the microtubule cytoskeleton has emerged as a relevant molecular substrate for the pervasive effects of alcohol. Several studies in rodent and human brain samples have shown that chronic alcohol exposure alters the abundance and acetylation of tubulin isotypes, as well as proteins known to regulate microtubule stability and dynamics, in the prefrontal cortex (PFC), hippocampus (Hipp), and ventral striatum [6–13]. An elegant demonstration of the functional significance of these changes was provided for collapsin response mediator protein-2 (CRMP-2), a microtubule-binding protein whose translation is increased in the nucleus accumbens following excessive alcohol consumption [6]. Both systemic CRMP-2 inhibition by lacosamide and local *Crmp2* knockdown by RNA interference decreased alcohol intake. Furthermore, genome-wide association studies have identified variants of the microtubule-associated protein tau gene (*MAPT*)associated with alcohol consumption in humans [14–16].

Interestingly, the regulation of brain microtubules is also altered in individuals with major depressive disorder and rodent models of chronic stress, and antidepressant treatments can reverse these changes [17–26]. The functional significance of microtubules for affect regulation is demonstrated by the antidepressant-like properties of MAP4343, a synthetic derivative of pregnenolone that promotes tubulin assembly via its interaction with microtubule-associated protein 2 (MAP2) [17, 22, 27, 28]. Given the role of negative affect in AUD, we hypothesized that this compound might reduce alcohol consumption driven by negative reinforcement.

Accordingly, in the present study, we tested the ability of acute and chronic administration of MAP4343 to reduce excessive alcohol drinking in a mouse model of ethanol intake escalation induced by chronic intermittent ethanol (CIE) vapor exposure [29]. We also examined the effect of chronic MAP4343 treatment on behavioral and hormonal indices of affective disturbance during CIE withdrawal. Furthermore, we analyzed the effects of CIE withdrawal on tubulin post-translational modifications (PTMs) known to alter the structure and function of microtubules [30].

## Materials and methods

### Animals

Male C57BL/6J mice were obtained from Scripps Research rodent breeding colony. Sani-Chips (Envigo) were used as bedding substrate. Food (Teklad LM-485, Envigo) and water (reverse osmosis purified) were available ad libitum except during affective and locomotor testing. Mice were maintained on a 12 h/12 h light/dark cycle and behavioral testing was performed during the dark phase. All procedures were carried out in accordance with the National Institute of Health Guide for the Care and Use of Laboratory Animals and were approved by Scripps Research Institutional Animal Care and Use Committee.

### Drug

MAP4343 was provided by MAPREG (Kremlin Bicêtre, France) as a micronized powder and was stored at room temperature, protected from light. A suspension was prepared in sesame oil (Sigma-Aldrich, S3547) at 20 mg/mL, vortexed, bath sonicated (2 pulses of 15 s at 100% power) and incubated at 35 °C for 30 min immediately prior to injection. MAP4343 was administered subcutaneously in a volume of 1–2 mL/kg using a 25G needle.

### Experimental design

#### Cohort 1—effect of MAP4343 in alcohol-drinking mice

**Fig. 1:**
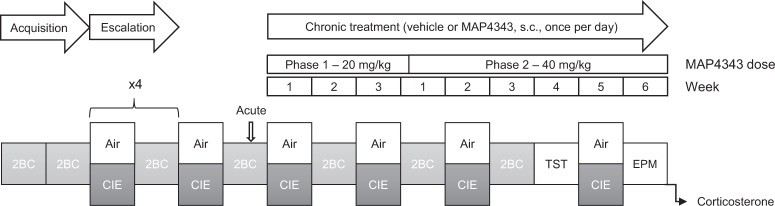
Experimental timeline. Male C57BL/6J mice were subjected to weeks of two-bottle choice (2BC) ethanol drinking (light gray boxes, 2 h per day, 5 days per week), alternated with weeks of Air (white boxes) or chronic intermittent ethanol (CIE) vapor inhalation (dark gray boxes, 4 × 16 h per week). The acute effect of MAP4343 on ethanol intake (20 mg/kg, s.c., 1 h before 2BC) was tested. Then, MAP4343 was administered daily (at least 2 h away from ethanol exposure) for 9 consecutive weeks, first at 20 mg/kg (22 days) then at 40 mg/kg. During this period, mice were tested for ethanol intake (2BC), immobility in the tail suspension test (TST), exploration of an elevated plus maze (EPM), and plasma corticosterone levels.

Thirty-six mice were transferred to our facility at 9 weeks of age. They were acclimated for 6 days, then single-housed, and testing started 3 days later. The mice remained single-housed throughout the duration of the experiment. The experimental timeline is shown in Fig. 1. Twenty-eight mice were subjected to seven sessions of two-bottle choice (2BC) alcohol drinking, then split into two groups of equivalent baseline ethanol intake that were then exposed to either CIE vapor inhalation to induce an escalation of voluntary ethanol intake (CIE-2BC, *n* = 16), or air only as a control condition (Air-2BC, *n* = 12). The remaining eight mice had access to two bottles of water during 2BC sessions and inhaled air only (Air-water), they served as alcohol-naive controls for affective and hormonal testing. Two CIE-2BC mice died over the course of the experiment.

**Fig. 2:**
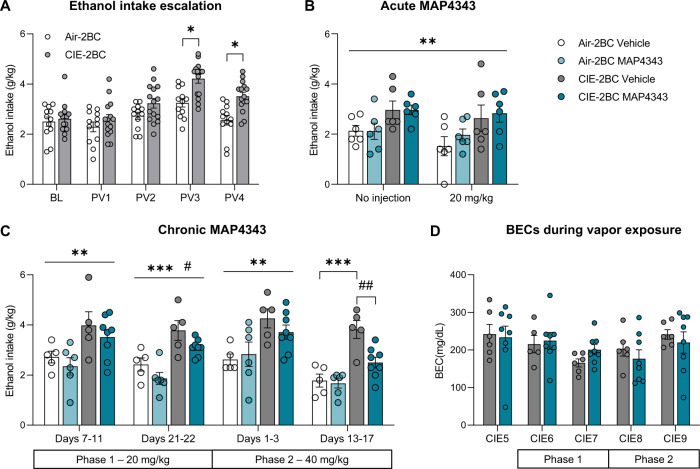
Chronic MAP4343 reduces alcohol drinking. **A**–**C** Ethanol intake in 2-h two-bottle choice (2BC) sessions. **A** Before MAP4343 treatment. **B** Upon acute administration of MAP4343 (20 mg/kg, s.c., 1 h before 2BC). **C** During chronic administration of MAP4343 (Phase 1: 20 mg/kg, Phase 2: 40 mg/kg, s.c., once per day, at least 18 h prior to 2BC sessions). *p < 0.05; **p < 0.01; ***p < 0.001, Air-2BC vs. CIE-2BC (**A**), main effect of CIE (**B** and **C**, lines) or Air-2BC Vehicle vs. CIE-2BC Vehicle (**C**, bracket); ^##^*p* < 0.01, CIE-2BC Vehicle vs. CIE-2BC MAP4343 (**C**). **D** Blood ethanol concentrations during vapor exposure, before (CIE5) and during chronic MAP4343 treatment. Data are shown as mean ± s.e.m.

After five rounds of CIE/2BC alternation, Air-2BC and CIE-2BC mice were further split into two treatment subgroups that received either vehicle or MAP4343 injections. The acute effect of MAP4343 was first tested by injecting vehicle or 20 mg/kg MAP4343 1 h prior to 2BC, 7 days after CIE week 5. Ethanol intake on the previous day is shown for reference in Fig. 2B (no injection was administered that day).

Chronic treatment was then started the following week (CIE week 6) by injecting a vehicle or MAP4343 once a day. A dose of 20 mg/kg was used for the first 22 days (phase 1) and then increased to 40 mg/kg until the end of the experiment (phase 2). The mice were injected 4–6 h into the dark phase, i.e., 2–4 h prior to vapor onset on CIE weeks and 2–4 h after alcohol drinking on 2BC weeks. A between-subject design was used for both the acute and chronic phases of the experiment and each mouse received the same treatment in all phases. All Air-water mice received vehicle injections.

The alternation of CIE/2BC was continued until post-vapor (PV) week 8 (third week of phase 2 treatment); 2BC sessions were then suspended and affective behaviors were examined. Daily vehicle/MAP4343 injections were administered after behavioral tests, such that 18–22 h elapsed between a given injection and subsequent testing. The tail suspension test (TST) was conducted during the fourth week of phase 2 treatment, 11 days after CIE week 8 and 4 days after the last 2BC session. The mice were also subjected to the grooming test [31], social investigation test [32], and digging test [33] 10 days, 13 days, and 17 days after CIE week 8, respectively, but there was no significant main effect or interaction of Alcohol history and Treatment in these tests (Supplementary Fig. 1). The elevated plus maze (EPM) test was conducted on the sixth week of phase 2 treatment, 4 days after CIE week 9 (no intervening 2BC). Mice were euthanized 2 days later, 1–5 h after dark phase onset, 19–23 h after vehicle/MAP4343 injection. Trunk blood was collected to quantify plasma corticosterone.

#### Cohort 2—effect of MAP4343 in alcohol-naive mice

Eighteen mice were single-housed at 9 weeks of age. Two weeks later, they were split into two groups of equivalent average body weight and assigned to vehicle (*n* = 9) or 40 mg/kg MAP4343 (*n* = 9) treatment. The experimental timeline is shown in Fig. 4A. The timing of daily injections was identical to that used in Cohort 1. Body weights were measured once a week. At the end of the third treatment week, home cage food and water intake were measured over two consecutive 24-h periods and averaged for analysis. On the fourth treatment week, saccharin intake was measured on 3 consecutive days in 2-h 2BC sessions started at the dark onset. Intake was similar across days and was therefore averaged for analysis. The TST was conducted three days later. During the fifth treatment week, mice were habituated to the locomotor activity recording setup on 2 consecutive days; on the following day, data were collected for 1 h prior to daily vehicle/MAP4343 administration (i.e., 20–24 h after the previous injection); on the next day, data were collected for 1 h immediately after daily vehicle/MAP4343 administration. On the sixth treatment week, the EPM test was conducted. Two days later, MAP4343-treated mice were split into two subgroups of equivalent average body weight and assigned to one of two blood sampling timepoints. Blood was collected either 1 h after MAP4343 injection (*n* = 4) or 20 h later (*n* = 5) to quantify plasma MAP4343 levels.

#### Cohort 3—plasma MAP4343 levels after acute administration

Twelve mice were single-housed at 10 weeks of age. Two months later, they were randomly assigned to the vehicle or MAP4343 treatment and to 1-h or 20-h sampling timepoint (*n* = 3 per condition). Blood was collected to quantify plasma MAP4343 levels.

#### Cohort 4—effect of CIE on tubulin

A cohort of Air-2BC (*n* = 9) and CIE-2BC (*n* = 7) mice was prepared for molecular analysis of brain samples (Fig. 5). They were exposed to a total of 9 weeks of CIE, alternated with weeks of 2BC, and brains were collected 7 days after last vapor exposure (no intervening 2BC).

### Behavioral testing

Drinking sessions, vapor exposure, TST, and EPM were conducted as in [34]. Details are provided in Supplementary Methods. Briefly, ethanol intake escalation was induced by alternating weeks of voluntary alcohol drinking during limited access 2BC sessions with weeks of forced CIE exposure via vapor inhalation. During 2BC weeks, Air-2BC and CIE-2BC mice were given access to ethanol (15% v:v) Monday-Friday for 2 h starting at the beginning of the dark phase. During CIE weeks, CIE-2BC mice were exposed to 4 cycles (Monday to Friday) of 16 h ethanol vapor inhalation/8-h air inhalation followed by 72 h withdrawal (Friday–Monday). Blood ethanol concentrations (BEC) were measured weekly using gas chromatography (GC) and flame ionization detection (Agilent 7820A).

Saccharin 2BC sessions used a concentration of 0.02% saccharin to approximate the volume of solution consumed in ethanol 2BC sessions.

For locomotor activity, mice were placed in individual cages lined with bedding. The same cage was used across days for each mouse. The horizontal motion was videotracked and analyzed using ANY-maze software (Stoelting).

### Corticosterone assay

Mice were euthanized by cervical dislocation immediately followed by decapitation. Trunk blood was collected in an EDTA-coated tube. Plasma was separated by centrifugation at 600 g for 15 min at 4 °C and stored at −80 °C. Corticosterone levels were measured in duplicates using the Corticosterone Enzyme Immunoassay Kit (Arbor Assays, Ann Arbor, MI) according to the manufacturer’s instructions.

### MAP4343 quantitation

Submandibular blood was collected in EDTA-coated tubes under isoflurane anesthesia. Plasma was separated by centrifugation at 600 g for 15 min at 4 °C and stored at −80 °C. A volume of 250 μL ice-cold acetonitrile (ACN):MeOH (50:50) was added to 50 μL of serum, vortexed for 1 min, kept at 4 °C for 30 min, and centrifuged at 17,000 g for 15 min. The supernatant was evaporated. A volume of 100 μL of ACN:MeOH was added, sonicated for 10 min, centrifuged at 17,000 g for 10 min, then transferred to an autosampler tube. Standards (10–500 ng/μL) were spiked in blank plasma and extracted using the same method. Quantitation was performed using an Agilent 8890 GC system coupled to an Agilent 5977B mass spectrometer. A volume of 1 μL was injected using a splitless injection technique onto a CP-FFAP CB column (Agilent, CP7485) at a flowrate of 2 mL/min. The initial temperature was 70 °C, held for 5 min, then ramped to 250 °C at 25 °C/min and held for 12 min. The front injector port and transfer line were set at 250 °C. The major fragment ion m/z = 298 was monitored for quantitation. Data were processed using Agilent MassHunter Quantitative Analysis software. Representative spectra and calibration curves are shown in Supplementary Fig. 2.

### Immunoblotting

#### Sample collection

Mice were euthanized by cervical dislocation and brains were immediately snap-frozen in isopentane. Micropunches of medial PFC (mPFC), insular cortex (Ins), amygdala (Amg), and Hipp were collected in a cryostat from 200-μm coronal sections using a 1-mm tissue corer (Fine Science Tools, 18035-01), as shown in Fig. 5B. The following anteroposterior coordinates (mm from bregma) were used for reference [35]: mPFC, +1.98 to +1.38; Ins, +0.98 to −0.02; Amg, −1.22 to −1.82; Hipp, −1.62 to −2.42. We selected these brain regions because chronic stress was previously shown to alter tubulin PTMs in the Hipp, Amg and PFC [17, 18, 22, 24], while fear conditioning alters stathmin and tubulin PTMs in the dorsal Hipp and Ins [36–38].

#### Tissue homogenization

Punches were thawed on wet ice for 5 min, then ice-cold buffer [10 mM Tris-Cl pH 8, 100 mM NaCl, 1 mM EDTA pH 8, and the following inhibitors at 1X: Protease Inhibitor Cocktail, Phosphatase Inhibitor Cocktail 2, and Phosphatase Inhibitor Cocktail 3 (respective catalog numbers: P8340, P5726, and P0044; Sigma-Aldrich)] was added to the tubes containing the punches. Next, approximately 200 mg of 0.5 mm zirconium oxide beads (Next Advance) were added and the punches were homogenized using a Bullet Blender Storm 24 tissue homogenizer (Next Advance) at speed 12 for 2 min at 4 °C. An equal volume of another ice-cold buffer [10 mM Tris-Cl pH 8, 100 mM NaCl, 1 mM EDTA pH 8, 4% (v/v) Triton X-100, 2% (v/v) SDS, and 1X inhibitors] was added and the homogenates were incubated end over end for 15 min at 4 °C using a tube revolver/rotator (ThermoFisher Scientific). The homogenates were transferred to new tubes, then protein concentrations were determined using the Pierce BCA Protein Assay Kit (ThermoFisher Scientific) before the samples were diluted in 4× Laemmli Sample Buffer with 2-mercaptoethanol (Bio-Rad). Samples were stored at −80 °C until immunoblotting.

#### Immunoblotting

The diluted samples were thawed to room temperature, heated at 95 °C for 5 min, then cooled back to room temperature. Next, a volume corresponding to 5 μg of protein from each brain region was loaded into Any kD Mini-PROTEAN TGX Stain-Free Protein Gels (Bio-Rad) and electrophoresed at 200 V using a Mini-PROTEAN Tetra Cell electrophoresis apparatus (Bio-Rad). The proteins were transferred to a PVDF Immobilon-P membrane (Merck Millipore) using a Trans-Blot Turbo Transfer System (Bio-Rad) running at 30 V and 1 A for 30 min. After the transfer, the membranes were rinsed 2 × 5 min in TBS (50 mM Tris-Cl pH 8 and 50 mM NaCl), dried for 1 h at room temperature, then stored at 4 °C.

Membranes were reactivated in 100% methanol, rinsed 3 × 5 min in TBST (50 mM Tris-Cl pH 8, 50 mM NaCl, and 0.1% Tween 20), then blocked for 1 h at room temperature in blocking buffer [TBST with 5% (w/v) blotting grade nonfat dry milk (Bio-Rad)]. Next, the membranes were incubated overnight at 4 °C in blocking buffer containing each primary antibody: α-tubulin, 1:5000 (Sigma-Aldrich, T9026), acetylated α-tubulin, 1:2000 (Sigma-Aldrich, T6793), tyrosinated α-tubulin, 1:1000 (Sigma-Aldrich, T9028), detyrosinated α-tubulin, 1:1000 (EMD Millipore, AB3201), Δ2 α-tubulin, 1:1000 (EMD Millipore, AB3203), and polyglutamylated tubulin, 1:1000 (Sigma-Aldrich, T9822). Next, the membranes were washed at room temperature for 3 × 5 min with blocking buffer, incubated for 1 h at room temperature in blocking buffer containing a 1:20,000 dilution of anti-rabbit IgG (H+L) horseradish peroxidase-conjugated antibody (Bio-Rad #1706515) or anti-mouse IgG (H+L) horseradish peroxidase-conjugated antibody (Bio-Rad #1706516), then washed at room temperature for 3 × 5 min with TBST. Finally, membranes were bathed with 1 mL of SuperSignal West Pico Chemiluminescent Substrate (ThermoFisher Scientific) and imaged using a ChemiDoc MP System (Bio-Rad).

To quantify the amount of protein loaded in each lane, the blots were stained with a Coomassie solution [0.5% (w/v) Coomassie Brilliant Blue R-250 Dye, 50% (v/v) methanol, and 10% (v/v) acetic acid], destained with destaining solution [50% (v/v) methanol and 7% (v/v) acetic acid], then imaged using a ChemiDoc MP System (Bio-Rad), as described in [39].

#### Immunoblot data analysis

The density of immunolabeled bands, as well as Coomassie staining density in corresponding lanes, were acquired using Image Lab Software (version 5.2.1; Bio-Rad) with background subtraction enabled, using a disc size of 70 mm [40, 41]. The immunoblotting signal in each lane was normalized to Coomassie staining to adjust for variability in protein loading. Total protein staining is a superior loading control normalizer compared to a single housekeeping protein whose abundance may be affected by chronic ethanol exposure [40, 42, 43]. Since the samples were spread across two blots for each brain region (3–5 samples from each experimental subgroup per blot), the values were normalized to the average signal from Air samples loaded on the same blot to account for differences in transfer efficiency between blots. Normalized values for each condition were then averaged across blots and expressed as a percentage of Air means.

### Statistical analysis

Data were analyzed in Statistica 13.3 (TIBCO Software). Ethanol intake escalation was analyzed by 2 × 2 repeated measures (RM) analysis of variance (ANOVA). The acute effect of MAP4343 on ethanol intake was evaluated by 2 × 2 × 2 RM-ANOVA using Vapor (Air, CIE) and Treatment (vehicle, MAP4343) as between-subject factors and Day (previous, test) as within-subjects factor. Daily ethanol intake during chronic treatment is shown in Supplementary Fig. 3. For statistical analysis, these values were averaged in four blocks (days 7–11 and days 21–22 of Phase 1, and days 1–3 and 13–17 of Phase 2—the numbering of days refers to treatment days, the corresponding 2BC session was conducted on the following day) and first subjected to 4 × 2 × 2 RM-ANOVA. Separate 2 × 2 ANOVAs were then conducted for each period. A 5 × 2 RM-ANOVA was used to assess the effect of MAP4343 on BECs across time. For affective and hormonal assays, two types of ANOVAs were conducted. First, 2 × 2 ANOVAs were conducted to analyze the main effects of Vapor (Air, CIE) and Treatment (vehicle, MAP4343) and their interaction. Then, one-way ANOVAs were used to examine the effect of Alcohol history in vehicle-treated mice (one factor with three levels: Air-water, Air-2BC, and CIE-2BC). Significant interactions and main effects were followed up with Tukey’s post hoc tests. Two-tailed *t*-tests were used to analyze the effect of MAP4343 in alcohol-naive mice and the effect of CIE on tubulin PTMs. Data are reported as mean ± standard error of the mean (s.e.m.).

## Results

### Chronic MAP4343 reduces alcohol drinking

Significant escalation of 2BC ethanol intake was first established via CIE exposure (Figs. 1 and 2A). There was a significant Vapor × Time interaction (F_4,100_ = 5.3, *p* = 0.00065) and significant differences between Air-2BC and CIE-2BC mice were detected from post-vapor week 3 onwards (PV3: *p* = 0.011; PV4: *p* = 0.013).

The Air-2BC and CIE-2BC groups were then split into two Treatment subgroups. Administering MAP4343 (20 mg/kg) immediately prior to 2BC did not alter ethanol intake in either group compared to vehicle, but the stress of injection tended to reduce intake in all subgroups (Fig. 2B, Vapor effect: F_1,20_ = 9.0, *p* = 0.007; Treatment effect: F_1,20_ = 0.25, *p* = 0.62; Day effect: F_1,20_ = 4.1, *p* = 0.056; all interactions: F_1,20_ < 1.3, *p* > 0.28).

Chronic treatment was initiated during the following CIE week (Fig. 1). A daily dose of 20 mg/kg was used during the first 22 days (phase 1) and was then increased to 40 mg/kg for the remainder of the experiment (phase 2). We hypothesized that repeated MAP4343 administration would gradually but persistently correct CIE-induced microtubule anomalies and yield beneficial behavioral effects beyond the first hours following injection (i.e., slow-onset, long-lasting effect). Accordingly, daily injections were administered 2–4 h after 2BC sessions and at least 18 h elapsed before subsequent alcohol access. On CIE weeks, the mice were injected 2–4 h prior to vapor onset. In addition to testing the sustained effects of MAP4343, this design reduced the influence of injection stress on behavior and aimed to minimize potential metabolic interactions between high levels of MAP4343 and alcohol.

An effect of MAP4343 on ethanol intake emerged over time (Fig. 2C). RM-ANOVA revealed significant main effects of Vapor (F_1,20_ = 26.2, *p* = 0.00005) and Time (F_3,60_ = 14.5, *p* < 0.000001), as well as a trend for Treatment (F_1,20_ = 3.56, *p* = 0.074). Follow-up analyses conducted on each treatment period detected a significant main effect of Treatment after 21–22 days of 20 mg/kg daily administration (F_1,20_ = 6.56, *p* = 0.019) and a significant Vapor × Treatment interaction after 13–17 days of 40 mg/kg daily administration (F_1,20_ = 5.4, *p* = 0.03). In the latter week, MAP4343 reduced ethanol intake compared to vehicle in CIE-2BC mice (*p* = 0.007) but not in Air-2BC mice (*p* = 0.99). Furthermore, vehicle-treated CIE-2BC mice had a higher intake than their Air-2BC counterparts (*p* = 0.0004) while this difference was not significant in MAP4343-treated mice (*p* = 0.08).

BECs measured during CIE were not affected by MAP4343 at any stage of the chronic treatment (Fig. 2D; main effect of Treatment, F_1,11_ = 0.002, *p* = 0.97; main effect of Time, F_4,44_ = 2.1, *p* = 0.10; Treatment × Time interaction, F_4,44_ = 0.64, *p* = 0.64).

### Chronic MAP4343 interacts with CIE withdrawal to alter stress coping and corticosterone

**Fig. 3:**
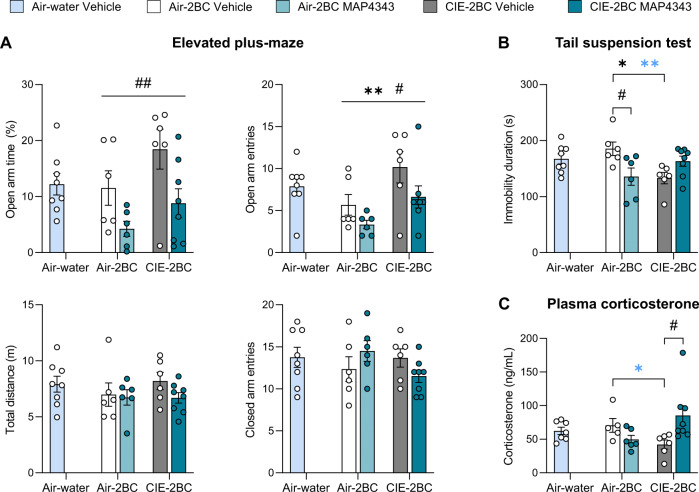
Chronic MAP4343 interacts with CIE withdrawal to alter stress coping and corticosterone. **A** Elevated plus maze. **B** Tail suspension test. **C** Plasma corticosterone levels. Two-way ANOVAs were used to examine the combined effects of chronic MAP4343 treatment (^#^) and CIE exposure (*), while one-way ANOVAs were used to examine the effect of Alcohol history in vehicle-treated mice (blue stars). Lines indicate main effects, brackets indicate post hoc comparisons. One symbol, *p* < 0.05; two symbols, *p* < 0.01. Data are shown as mean ± s.e.m.

For EPM, TST, and corticosterone measures, two-way ANOVAs were used to examine the combined effects of chronic MAP4343 treatment and CIE exposure, while one-way ANOVAs were used to examine the effect of Alcohol history in vehicle-treated mice (i.e., including Air-water Vehicle mice as a reference group to control for the effects of chronic 2BC drinking) (Fig. 3).

In the EPM (Fig. 3A), chronic MAP4343 reduced the time spent in open arms (F_1,22_ = 9.2, *p* = 0.006) and the number of entries into the open arms (F_1,22_ = 4.8, *p* = 0.039) without affecting the number of entries into the closed arms (F_1,22_ = 0.0, *p* = 1.0) or the total distance traveled (F_1,22_ = 1.4, *p* = 0.25). In contrast, CIE increased open-arm exploration (entries: F_1,22_ = 8.4, *p* = 0.008; time: F_1,22_ = 4.2, *p* = 0.051), also without altering closed-arm entries (F_1,22_ = 0.56, *p* = 0.46) or total distance (F_1,22_ = 0.63, *p* = 0.44). There was a trend for a Treatment × Vapor interaction for closed-arm entries (F_1,22_ = 3.8, *p* = 0.066) but none of the pairwise comparisons reached significance (*p*s > 0.23). All other interactions were insignificant (*p*s > 0.4). The main effect of Alcohol history in Vehicle mice did not reach significance for any of the measures (one-way ANOVAs, F_2,17_s < 2.5, *p*s > 0.1).

In the TST (Fig. 3B), analysis of the immobility duration revealed a significant Treatment × Vapor interaction (F_1,22_ = 11.8, *p* = 0.008). Post hoc comparisons showed that MAP4343 reduced immobility in Air-2BC mice (*p* = 0.036) and CIE reduced immobility in Vehicle mice (*p* = 0.026). In contrast, CIE-2BC MAP4343 mice did not differ from Air-2BC Vehicle mice (*p* = 0.51). Consistent with these results, there was a significant main effect of Alcohol history in Vehicle mice (F_2,17_ = 6.2, *p* = 0.0096), which was driven by shorter immobility durations in CIE-2BC mice compared to Air-2BC (*p* = 0.0083) and Air-water mice (trend, *p* = 0.067).

There was also a significant Treatment × Vapor interaction for plasma corticosterone levels (Fig. 3C, F_1,21_ = 7.9, *p* = 0.010). Post hoc comparisons indicated that chronic MAP4343 increased corticosterone levels selectively in CIE-2BC mice (*p* = 0.044). In contrast, CIE-2BC MAP4343 mice did not differ from Air-2BC Vehicle mice (*p* = 0.79). In Vehicle mice, there was a significant main effect of Alcohol history (F_2,15_ = 3.9, *p* = 0.044) resulting from significantly lower levels in CIE-2BC mice compared to Air-2BC mice (*p* = 0.046).

### Chronic MAP4343 has limited effects in alcohol-naive mice

**Fig. 4:**
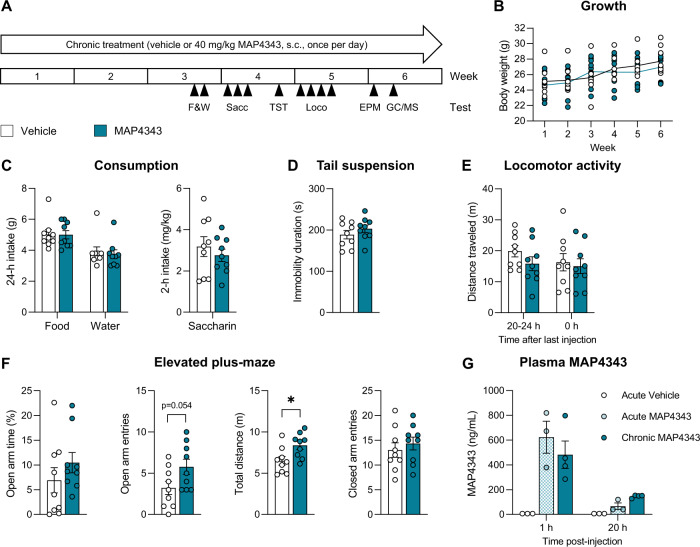
Chronic MAP4343 has limited effects in alcohol-naive mice. **A** Experimental timeline. Mice received daily injections of vehicle or 40 mg/kg MAP4343. Body weights were measured weekly (**B**). Mice were tested for food and water intake (F&W, **C**), saccharin consumption (Sacc, **C**), immobility in the tail suspension test (TST, **D**), locomotor activity (Loco, **E**), and exploration of an elevated plus maze (EPM, **F**). Plasma concentrations of MAP4343 were measured by gas chromatography/mass spectrometry (GC/MS, **G**). *p < 0.05, Vehicle vs. MAP4343. Data are shown as mean ± s.e.m.

To assist the interpretation of data obtained in alcohol-drinking mice, the effects of chronic 40 mg/kg MAP4343 treatment were examined in a separate cohort of C57BL/6J males (Fig. 4A). Body weights increased over time (Fig. 4B, F_5,80_ = 23.7, *p* < 0.000001) and there was no overall effect of MAP4343 on growth (F_1,16_ = 0.24, *p* = 0.63). There was a trend for a Time × Treatment interaction (F_5,80_ = 2.1, *p* = 0.070) but none of the pairwise comparisons reached significance (*p* > 0.99 on each week). MAP4343 did not impact the daily consumption of food (t_16_ = 0.05, *p* = 0.96) and water (t_16_ = 0.33, *p* = 0.74), nor limited access free-choice saccharin intake (t_16_ = 0.75, *p* = 0.47) (Fig. 4C). Immobility in the TST was also unchanged (Fig. 4D, t_16_ = −1.06, *p* = 0.31). There was no effect of MAP4343 on locomotor activity measured in a home-cage-like setting, either before (t_16_ = 1.39, *p* = 0.18) or after (t_16_ = 0.35, *p* = 0.73) administration of the daily injections (Fig. 4E). The total distance traveled in the EPM was higher in MAP4343-treated mice than in their vehicle-treated counterparts (Fig. 4F, t_16_ = −2.63, *p* = 0.018) but entries into closed arms did not increase significantly (t_16_ = −0.68, *p* = 0.51). MAP4343 tended to increase open-arm entries (t_16_ = −2.08, *p* = 0.054) but did not affect the time spent on open arms (t_16_ = −1.08, *p* = 0.30).

Given that significant effects of MAP4343 had been observed more than 18 h post-injection in alcohol-drinking mice, we examined whether MAP4343 was still present in the blood at that timepoint. We also tested whether the sesame oil vehicle may sustain continuous MAP4343 absorption through the formation of an s.c. depot over the course of chronic administration. To do so, we compared circulating concentrations of MAP4343 in mice receiving the compound for the first time vs. after 5 weeks of daily injections (Fig. 4G). As expected, no MAP4343 could be detected in vehicle-injected mice, confirming the selectivity of the analytical procedure. Plasma MAP4343 levels were similar between acutely and chronically treated mice (main effect of Regimen, F_1,10_ = 0.13, *p* = 0.73) and dropped more than 3-fold between 1 and 20 h post-injection (main effect of Timepoint, F_1,10_ = 27.1, *p* = 0.0004; Regimen × Timepoint interaction, F_1,10_ = 1.65, *p* = 0.23).

### Withdrawal from CIE-2BC reduces α-tubulin acetylation in the medial prefrontal cortex

**Fig. 5:**
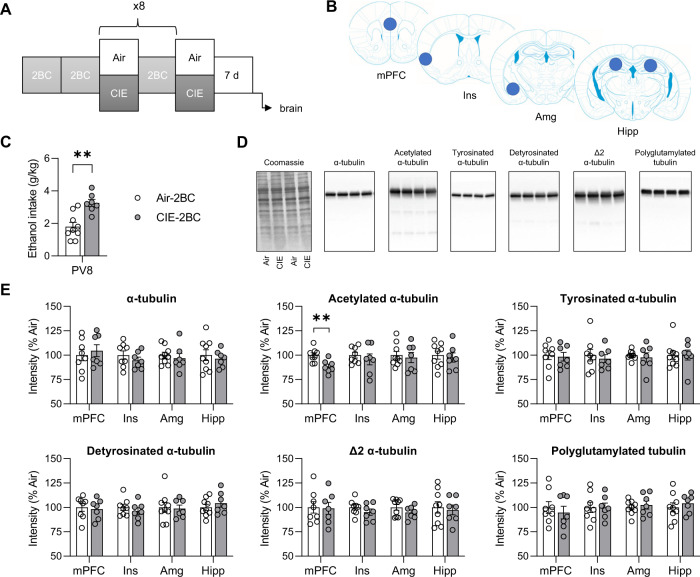
CIE withdrawal reduces α-tubulin acetylation in the medial prefrontal cortex. **A** Experimental timeline. Mice were exposed to 8 rounds of CIE/2BC alternation and brains were collected 7 days after an additional week of CIE. **B** Position of punches collected for each brain region: mPFC, medial prefrontal cortex; Ins, insular cortex; Amg, amygdala; Hipp, hippocampus. **C** Average ethanol intake during the last 2BC week. **D** Representative images of Coomassie staining and immunostaining of α-tubulin and tubulin post-translational modifications (PTM). Signal from the same 4 samples is shown in all images. **E** Quantification of protein and PTM abundance in the mPFC, Ins, Amg, and Hipp from Air (white bars) and CIE (gray bars) mice. Protein/PTM levels are expressed as a percentage of Air values. **p < 0.01, Air vs. CIE. Data are shown as mean ± s.e.m.

MAP4343 is thought to produce its behavioral effects via the modulation of microtubule assembly and stability [27, 28]. We therefore sought to determine whether the behavioral phenotypes of CIE withdrawal may be associated with changes in tubulin PTMs that are known to alter the biophysical properties of microtubules (acetylation of α-tubulin lysine 40) or their interactions with MAPs (removal of the C-terminal tyrosine and glutamate [Δ2] residues, or addition of negatively charged polyglutamate side chains) [30]. Accordingly, we collected brains from another cohort of C57BL/6J males subjected to CIE-2BC and withdrawn from CIE for 7 days (Fig. 5A) and analyzed four brain regions that contribute to excessive alcohol intake and show tubulin PTM alterations in models of depression and learning [17, 18, 22, 24, 36–38, 44] (Fig. 5B). As expected, CIE mice consumed significantly higher levels of ethanol than Air counterparts on their last 2BC week (Fig. 5C, t_14_ = −4.1, *p* = 0.001). In the mPFC, we detected a significant reduction in the levels of acetylated α-tubulin in CIE mice (Fig. 5D, E, t_13_ = 3.3, *p* = 0.006), while the total amount of α-tubulin and the other PTMs were not affected (*p*s > 0.5). The correlation between mPFC levels of acetylated α-tubulin and PV8 ethanol intake did not reach significance (*r*^2^ = 0.21, *p* = 0.087). Withdrawal from CIE had no significant effects in the Ins, Amg, or Hipp (Fig. 5D, E).

## Discussion

Our results indicate that chronic administration of MAP4343 reduces alcohol drinking in C57BL/6J male mice. A mild lowering of ethanol intake was observed in both moderate (Air-2BC) and excessive (CIE-2BC) alcohol drinkers after 3 weeks of daily treatment with 20 mg/kg MAP4343. Additional weeks of treatment with a daily dose of 40 mg/kg produced a more robust reduction that was selective for escalated ethanol intake in CIE-2BC mice. The effects of this treatment regimen on affective and hormonal endpoints were complex and interacted with those of alcohol. In the EPM, MAP4343 tended to increase open-arm exploration in alcohol-naive mice but reduced it in alcohol-withdrawn mice. In the TST, chronic MAP4343 reduced immobility in Air-2BC mice but tended to increase it in CIE-2BC mice. Likewise, chronic MAP4343 elevated plasma corticosterone levels in CIE-2BC mice but tended to reduce them in Air-2BC mice. Altogether, this pattern suggests that affective and/or hormonal effects of chronic MAP4343 do not directly mediate its effects on alcohol drinking. However, it resulted in a relative normalization of affective and hormonal endpoints compared to the vehicle-treated alcohol-naive mice that were tested in parallel with Air-2BC and CIE-2BC mice. We also show that CIE reduces α-tubulin acetylation in the mPFC, which suggests that mPFC microtubules may be less resistant to mechanical stress following a history of excessive alcohol exposure [45–47].

This is the first report of MAP4343 administration in mice. Experiments conducted by MAPREG had shown that, in adult C57BL/6J mice, MAP4343 reduces immobility in the forced swim test (FST) after 3 s.c. injections (24, 5 and 1 h before FST) at a dose of 20 mg/kg, but not 5 and 10 mg/kg (personal communication from Nicolas Froger). A single injection of 20 mg/kg 1 h prior to FST also significantly reduced immobility in adult Swiss mice (personal communication from Nicolas Froger). Similar results had been obtained in Sprague-Dawley rats at the lower doses of 4 and 10 mg/kg, but not 15 mg/kg [17]. Other studies conducted in rats had shown that daily s.c. administration of MAP4343 4–12 mg/kg for 4–10 days improves functional recovery from spinal cord injury [48] and produces anxiolytic-like and antidepressant-like effects following isolation rearing [17]. Based on these data, we reasoned that a single or repeated administration of 20 mg/kg MAP4343 might reduce alcohol drinking in C57BL/6J mice experiencing affective disturbances caused by repeated cycles of alcohol intoxication and withdrawal. Given that 20 mg/kg MAP4343 produced a modest and unstable effect on alcohol consumption after 3 weeks of daily administration (phase 1), we increased the dose to 40 mg/kg for subsequent testing (phase 2). The effect of MAP4343 was lost during the first days of treatment with this higher dose, further reflecting the poor stability of alcohol intake reduction achieved by the lower dose. In contrast, after two weeks of daily exposure to 40 mg/kg MAP4343, CIE-2BC mice consistently consumed 25–45% less alcohol than vehicle-treated counterparts throughout the entire 2BC week, while Air-2BC mice remained unaffected all along. This outcome may result from the cumulative effects of phase 1 and phase 2 dosing, and it is possible that a similar effect would have been observed with more prolonged treatment with the lower dose. Importantly, the effect of MAP4343 on alcohol drinking was likely not due to altered ethanol metabolism since BECs during vapor exposure were similar in vehicle-treated and MAP4343-treated mice throughout the experiment.

Future studies will be needed to determine the ability of chronic MAP4343 to reduce excessive alcohol drinking in females. Only male mice were included in the present study because our primary endpoint was escalated alcohol intake and, in our experience, CIE-induced drinking escalation is challenging to obtain in C57BL/6J females (e.g., [49], see also [50], but [51]), even though both sexes exhibit affective disturbances during CIE withdrawal [34, 49, 52]. A different model of alcohol drinking escalation, such as intermittent access (IA, [53]), could be used in females, although the neurobiological mechanisms driving escalation in the CIE-2BC and IA models do not necessarily overlap and may respond differentially to chronic MAP4343 administration. Alternatively, the affective effects of MAP4343 could be tested in females withdrawn for 15 days from long-term continuous access to voluntary alcohol consumption, a model that consistently yields depression-like behavior [54].

In contrast to the data mentioned above, we did not observe an effect of chronic MAP4343 treatment on depression-like behavior (TST) in alcohol-naive mice and there was only a trend for anxiolytic-like activity in the EPM. A major procedural difference is the timing of MAP4343 injections, which were administered shortly before testing in previous studies but more than 18 h earlier in our study. Our pharmacokinetic assessment indicated that MAP4343 is still present in the blood at the latter timepoint but at concentrations much lower than 1 h post-injection. Repeated administration did not significantly alter this pharmacokinetic profile. It is therefore possible that circulating levels of MAP4343 need to be above a specific threshold for antidepressant-like and anxiolytic-like activity to be detected. Importantly, this was not the case for alcohol intake, which was significantly reduced more than 18 h post-injection. Future studies will be needed to determine whether this sustained effect of repeated MAP4343 administration on alcohol drinking might persist for more than one day after treatment cessation.

In vehicle-treated mice, CIE increased EPM open-arm exploration and reduced TST immobility. These phenotypes are opposite to those reported in CIE-withdrawn rats [55, 56] but complement those reported by us and others in CIE-withdrawn C57BL/6J males [49, 52, 57] and highlight the complexity of interpreting affective readouts in rodents and validating the role of negative reinforcement in CIE-induced ethanol intake escalation. We had previously reported that EPM and TST behaviors were not affected by a history of CIE-2BC when tested 10–11 days and 19 days, respectively, after the last vapor exposure [49]. Here, we found that shorter withdrawal durations (4 days and 11 days, respectively) are associated with more frequent visits to the EPM open arms and less immobility in the TST, when compared to Air-2BC counterparts. Interpreting these observations in the context of anxiety yields contradictory conclusions as increased open-arm exploration in the EPM would reflect an anxiolytic-like effect of CIE withdrawal [58], while reduced immobility in the TST would reflect an anxiogenic-like effect of CIE withdrawal [59]. Moreover, the reduced EPM open-arm exploration induced by MAP4343 in alcohol-drinking mice raises the possibility that alcohol intake reduction may result from increased anxiety, rather than from an alleviation of negative reinforcement. However, an alternative interpretation for these phenotypes is that CIE withdrawal promotes behavioral disinhibition and active stress coping strategies rather than altering anxiety states (see [60, 61]). In contrast to behavioral measures, the reduced plasma corticosterone levels we observed in vehicle-treated CIE-2BC mice vs. Air-2BC mice are consistent with the effect of CIE in rats, as peak corticosterone levels were also lower in ethanol self-administering rats withdrawn from CIE for 9 days compared to Air-exposed counterparts [62].

While our findings do not support the role of negative reinforcement in CIE-induced alcohol intake escalation and its reversal by MAP4343, an interesting pattern emerges if we refrain from assigning a subjective meaning to EPM and TST measures and simply treat them as objective indices of change. Notably, the combination of MAP4343 treatment and CIE exposure yielded open-arm exploration levels similar to those of vehicle-treated, ethanol-naive mice. Likewise, despite MAP4343 treatment and CIE exposure similarly reducing immobility in the TST, their combination paradoxically normalized the phenotype of CIE-2BC mice, which behaved similarly to control mice. Plasma corticosterone levels followed a similar pattern across groups, with chronic MAP4343 producing seemingly opposite effects in Air-2BC and CIE-2BC mice, and reverting CIE-2BC mice to control levels. Altogether, it is striking that chronic MAP4343 corrected three independent phenotypes associated with withdrawal from CIE-2BC, even if the biological meaning of these phenotypes remains difficult to interpret.

Neuroactive steroids are known to influence alcohol drinking [63]. Most relevant to our study, chronic treatment with supraphysiological doses of pregnenolone reduces stress- and alcohol cue-induced craving in individuals with AUD [64]. While MAP4343 retains the ability of pregnenolone to bind MAP2 and promote tubulin polymerization, it does not get converted into pregnenolone and thus does not produce derivatives known to act on ionotropic receptors [27, 28, 65]. Accordingly, microtubules are expected to mediate the behavioral effects of MAP4343.

We found that withdrawal from CIE-2BC reduced levels of α-tubulin acetylation in the mPFC but did not affect total levels of α-tubulin, nor α-tubulin tyrosination, C-terminal truncation or polyglutamylation. In contrast, α-tubulin acetylation was unaltered in the Ins, Amg, and Hipp. Microtubule acetylation promotes kinesin-mediated cargo trafficking in neurites [66]. Consistent with this role, reduced microtubule acetylation is known to cause axonal transport defects associated with multiple neurodegenerative disorders [67–72]. Our results therefore suggest that CIE withdrawal may affect axonal transport in the mPFC, which could impact neurotransmission locally and in regions innervated by the mPFC. More recently, α-tubulin acetylation was shown to increase the flexibility of microtubules by weakening lateral interactions between protofilaments, making them more resistant to mechanical stress [45]. Furthermore, acetylation hotspots accumulate at stress-induced cracks in the microtubule lattice, which is thought to facilitate self-repair and explains the enrichment of this PTM in long-lived microtubules [46, 47, 73]. Accordingly, reduced acetylation of mPFC microtubules could reflect their premature mechanical ageing in CIE-withdrawn mice. Paradoxically, our results are at odds with two clinical studies that identified reduced total levels of α-tubulin and a significant increase in α-tubulin acetylation in the PFC of AUD subjects [7, 12]. A possible explanation for this discrepancy is that some of the AUD subjects were intoxicated at the time of death, while our mice were withdrawn for 7 days. Moreover, histological analysis revealed abnormalities in the cytoplasm appearance of PFC cells from AUD subjects, suggesting a loss of cellular integrity that may not be achieved in the CIE-2BC mouse model [7]. Relevant to the hypothesis that microtubule hypoacetylation might be specific to the withdrawal state, reduced tubulin acetylation was also observed in PFC tissue from depressed subjects, although a significant change was only detected in the membrane fraction, and lipid rafts in particular [26].

While our results do not establish a mechanistic link between microtubule acetylation and the ability of MAP4343 to reduce alcohol drinking, one may speculate that the interaction of MAP4343 with MAP2 could contribute to reversing the functional consequences of microtubule hypoacetylation in CIE-exposed mice. Importantly, this working hypothesis does not require that chronic MAP4343 directly affects tubulin acetylation state, as the functional consequences of hypoacetylation may be countered by heterologous mechanisms, for instance via the modulation of protein-protein interactions.

In conclusion, we show that chronic treatment with the pregnenolone derivative MAP4343 can reduce excessive alcohol drinking in C57BL/6J male mice under experimental conditions that produce tubulin hypoacetylation in the mPFC. These findings support the relevance of microtubules as a therapeutic target for the treatment of AUD.

## Supplementary information

Supplementary Material
