# Supplementary material for: Chronic MAP4343 reverses escalated alcohol drinking in a mouse model of alcohol use disorder
Source: Neuropsychopharmacology. 2023 Jan 20;48(5):821–30. doi: 10.1038/s41386-023-01529-z (PMC10066354; doi:10.1038/s41386-023-01529-z)
Supplement: Supplementary file 1 — Supplementary Material [file 41386_2023_1529_MOESM1_ESM.pdf]

Supplementary Methods

Ethanol intake escalation

Ethanol intake escalation was induced by alternating weeks of voluntary alcohol drinking during limited-access 2BC sessions with weeks of forced CIE exposure via vapor inhalation.

During 2BC weeks, Air-2BC and CIE-2BC mice were given access to ethanol Monday-Friday for 2 h starting at the beginning of the dark phase. During 2BC sessions, the home cage water bottle was replaced with two 50-mL bottles, one containing ethanol (15% v:v) and the other containing water. The two bottles were weighed before and after each 2-h session. Fluid loss during handling was estimated by control bottles (empty cage) to calculate ethanol intake. Air-water mice were given access to two bottles of water.

During CIE weeks, CIE-2BC mice were exposed to 4 cycles (Monday to Friday) of 16 h ethanol vapor inhalation/8-h air inhalation followed by 72 h withdrawal (Friday-Monday). At the onset of each ethanol vapor inhalation period, animals received an intraperitoneal (i.p) injection of ethanol (1.5 g/kg, PHARMCO-AAPER, 111000200) to initiate intoxication and pyrazole (1 mmol/kg, Sigma-Aldrich, P56607) to normalize ethanol clearance rate between individual mice (vehicle: saline). Blood ethanol concentrations were measured on a weekly basis using gas chromatography and flame ionization detection (Agilent 7820A). Air-2BC and Air-water mice remained in their home cages and were injected with pyrazole only.

Tail suspension test

The procedure was conducted as described by Can et al. [1]. The mice were suspended by their tails using adhesive tape wrapped around the tail approximately 2 cm from the tip and affixed to shelving. Prior to taping, the tail was inserted in a clear hollow cylinder (3.5-cm length, 1-cm diameter, 1 g) to prevent tail climbing behavior. The test lasted 6 min and the total duration of immobility was recorded. Testing was conducted under red light.

Elevated plus-maze

The apparatus was made of matte grey acrylic and consisted of two opposite open arms (30 cm length × 5 cm width), with a 0.3 cm lip, and two enclosed arms of the same size, with 15 cm high walls made of translucent acrylic. The runways were elevated 30 cm above the ground. Testing began by placing an animal on the central platform of the maze facing an open arm. The test lasted 5 min and the maze was cleaned between subjects. The following measures were recorded using the ANY-maze (Stoelting Co.) behavioral tracking system: total distance traveled, time spent and number of entries in the open and closed arms. Testing was conducted under red light.

Supplementary Reference

1. Can A, Dao DT, Terrillion CE, Piantadosi SC, Bhat S, Gould TD. The tail suspension test. J Vis Exp. 2012; (59):e3769.

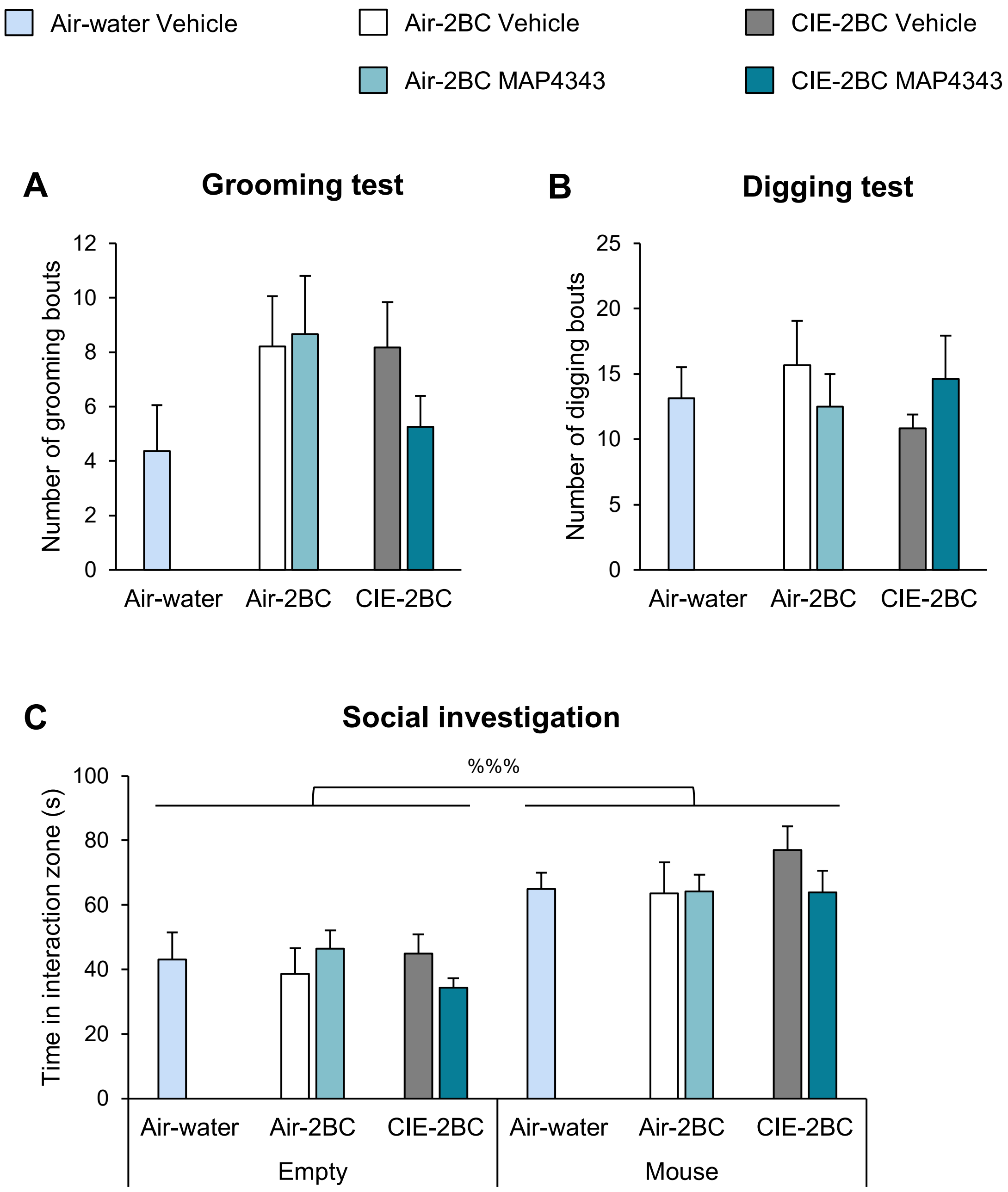

Supplementary Figure 1. Additional affective behaviors. There was no effect of alcohol exposure history or chronic MAP4343 treatment in the grooming test (**A**), digging test (**B**), and social investigation test (**C**). %%%, p<0.001, main effect of stranger mouse (RM-ANOVA). Data are shown as mean ± s.e.m.

A

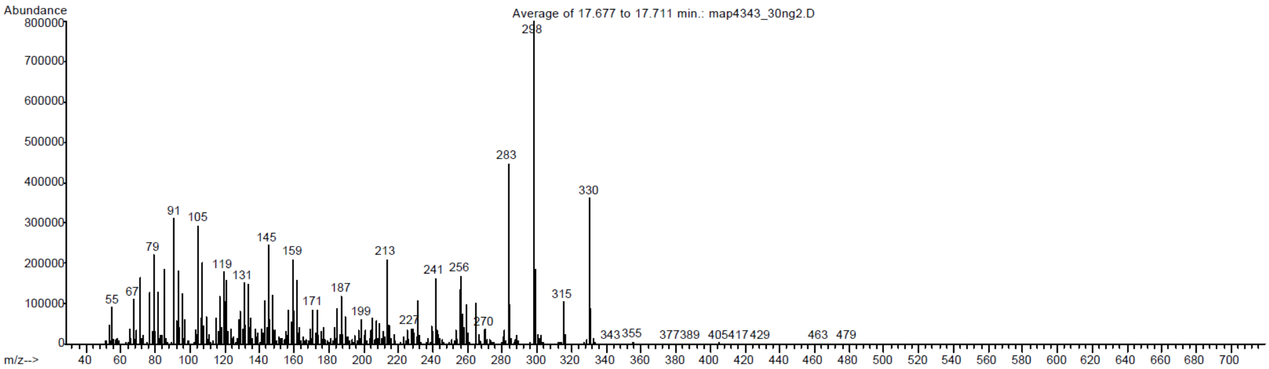

B

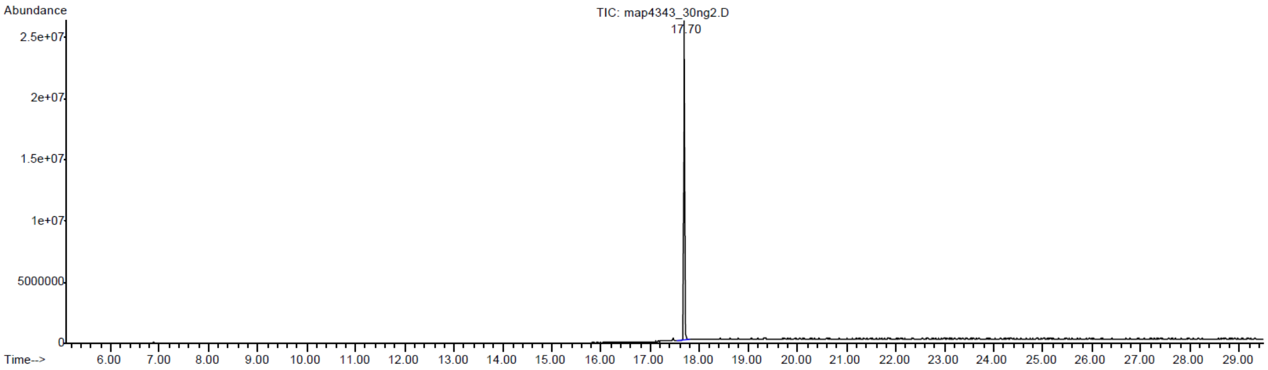

C

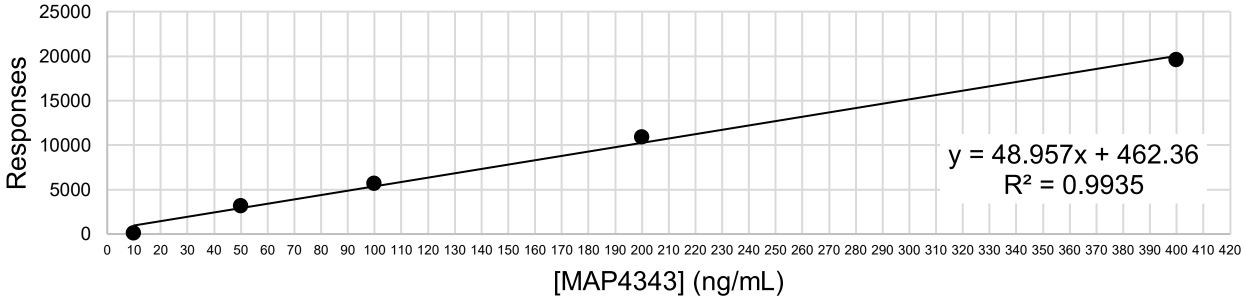

Supplementary Figure 2. MAP4343 quantification. A. Full MS scan of MAP4343 standard at 30 ng/µL. **B.** The major fragment ion m/z=298 ion was monitored for quantitation. **C.** Representative calibration curve used for the quantitation of MAP4343 plasma levels in experimental mice. Lower and upper limits of quantitation were 10 and 500 ng/mL, respectively.

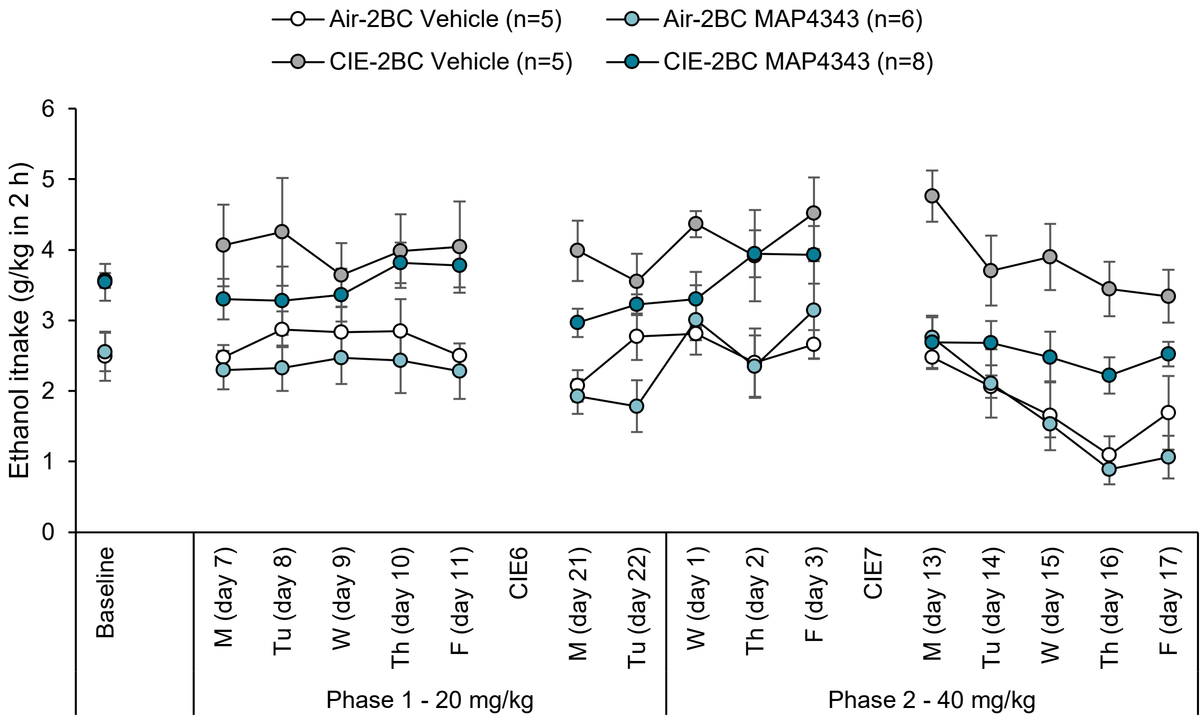

Supplementary Figure 3. Daily ethanol intake during chronic MAP4343 treatment. Data are shown as mean ± s.e.m.
